# Direct vs. Microclimate-Driven Effects of Tree Species Diversity on Litter Decomposition in Young Subtropical Forest Stands

**DOI:** 10.1371/journal.pone.0160569

**Published:** 2016-08-04

**Authors:** Katrin N. Seidelmann, Michael Scherer-Lorenzen, Pascal A. Niklaus

**Affiliations:** 1 Geobotany, Faculty of Biology, University of Freiburg, Freiburg, Germany; 2 Department of Evolutionary Biology and Environmental Studies, University of Zurich, Zürich, Switzerland; Helmholtz Centre for Environmental Research (UFZ), GERMANY

## Abstract

Effects of tree species diversity on decomposition can operate via a multitude of mechanism, including alterations of microclimate by the forest canopy. Studying such effects in natural settings is complicated by the fact that topography also affects microclimate and thus decomposition, so that effects of diversity are more difficult to isolate. Here, we quantified decomposition rates of standard litter in young subtropical forest stands, separating effects of canopy tree species richness and topography, and quantifying their direct and micro-climate-mediated components. Our litterbag study was carried out at two experimental sites of a biodiversity-ecosystem functioning field experiment in south-east China (BEF-China). The field sites display strong topographical heterogeneity and were planted with tree communities ranging from monocultures to mixtures of 24 native subtropical tree species. Litter bags filled with senescent leaves of three native tree species were placed from Nov. 2011 to Oct. 2012 on 134 plots along the tree species diversity gradient. Topographic features were measured for all and microclimate in a subset of plots. Stand species richness, topography and microclimate explained important fractions of the variations in litter decomposition rates, with diversity and topographic effects in part mediated by microclimatic changes. Tree stands were 2–3 years old, but nevertheless tree species diversity explained more variation (54.3%) in decomposition than topography (7.7%). Tree species richness slowed litter decomposition, an effect that slightly depended on litter species identity. A large part of the variance in decomposition was explained by tree species composition, with the presence of three tree species playing a significant role. Microclimate explained 31.4% of the variance in decomposition, and was related to lower soil moisture. Within this microclimate effect, species diversity (without composition) explained 8.9% and topography 34.4% of variance. Topography mainly affected diurnal temperature amplitudes by varying incident solar radiation.

## Introduction

**Fig 1:**
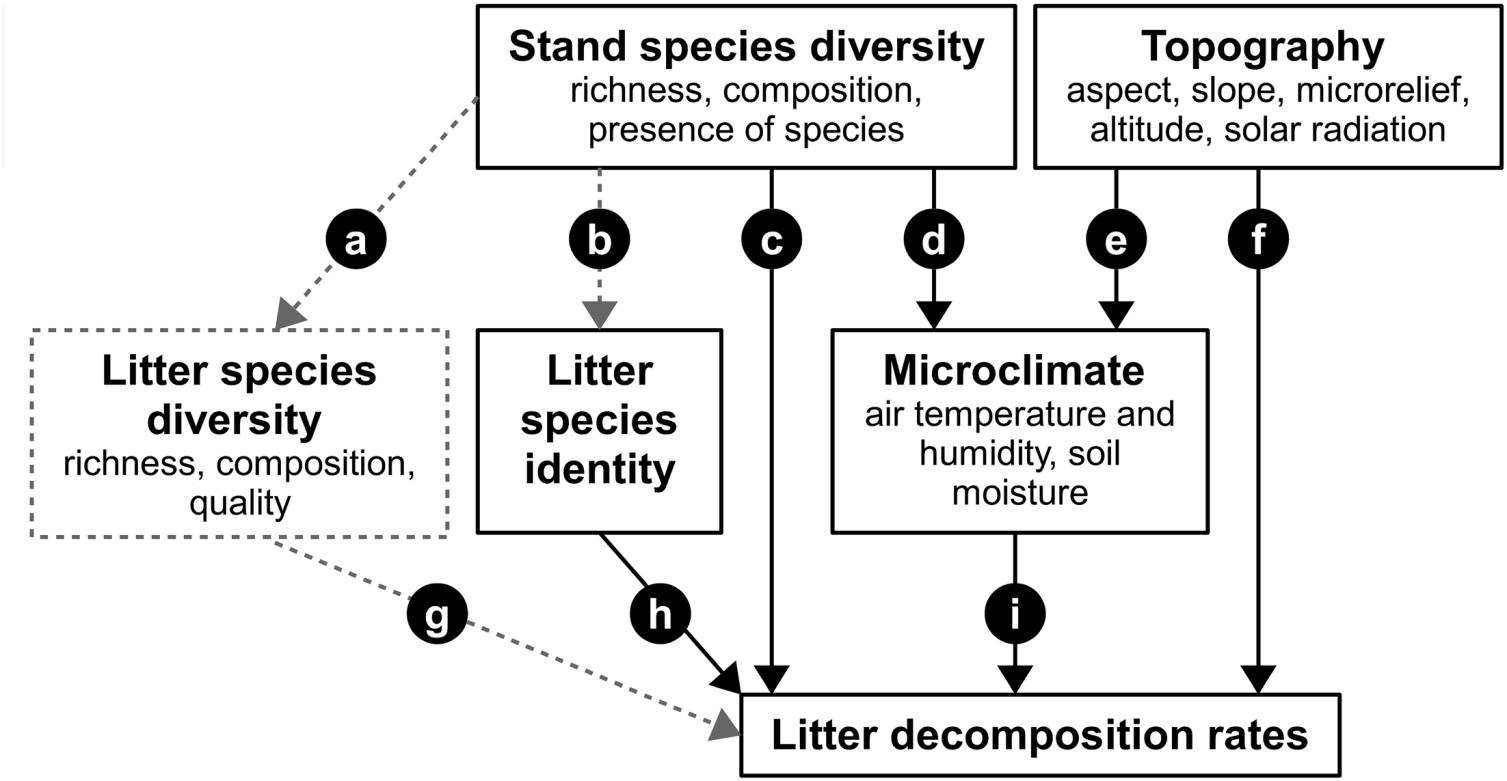
Components of stand species diversity and topography which likely affect litter decomposition rates either directly or via microclimate. Concept variables are bold typed with measured indicators underneath. Arrows indicate direct and indirect effects of stand species diversity (a-d, g-i) and site topography (e, f, i) on litter decomposition rates described in the main text. Effects not measured in this study are marked with dashed lines.

The degradation of plant litter is a key process in terrestrial global carbon (C) and nutrient cycling. Plant litter provides the main supply of energy to soil fauna and micro-organisms and shapes interactions between plant and soil communities [1,2]. Litter decomposition rates depend on a range of biotic (e.g. species identity and diversity of plants, litter and decomposers) and abiotic factors (e.g. temperature, moisture and radiation). However, quantifying such effects in natural settings is complicated because these factors are strongly interconnected and often act via a range of direct and indirect pathways (Fig 1). Effects of microclimatic conditions and substrate quality have been studies in detail. However, less is known on whether and how the tree species diversity of forest stands affects leaf litter decomposition rates through mechanisms other than the leaf litter composition.

Research of the past decades has provided ample evidence that plant species diversity (i.e. species richness and composition) affects ecosystem functioning, in particular primary production [3]. Plant community composition can not only affect the amount of litter produced, but also the composition of leaf litter and its chemical and morphological characteristics [2] (Fig 1a and 1b). Indeed, litter of different plant species decomposes at very different rates, depending on leaf chemical (e.g. nitrogen and lignin contents [4,5]) and morphological properties (e.g. leaf mass per area [6], thickness or tensile strength [7]) that stimulate or inhibit microorganisms [8] (Fig 1h). In addition, leaf litter decomposition studies have shown that mixing litter of different species often leads to non-additive effects, i.e. decomposition can increase or decrease with the number of plant species contained in mixed litter samples (reviewed by [9,10]; Fig 1g). In the majority of cases, however, positive mixing effects with accelerated decomposition prevail, which have been attributed to non-additive effects originating from fungi-driven nutrient transfer from high-quality litter to low-quality recalcitrant litter, and to higher habitat and food diversity for soil fauna [11]. While litter diversity is important for decomposition processes, variation in canopy species traits, in particular in seasonality and architecture, can alter light distribution, rainfall interception, stem flow and evapotranspiration [12,13] (see Fig 1d). The resulting effects on microclimate, in particular on air and soil temperature and humidity (e.g. [14–16]), potentially also affect litter decomposition rates through mechanisms that are independent of litter species composition (e.g. [17]; Fig 1i). However, comparatively few experiments have tested for such effects so far [18]. Most of these studies (e.g. [2,19–21]) did not find a relation between stand species richness and decomposition rates, but there is evidence that canopy and litter species composition interact (e.g. [11,22]; Fig 1c); for example, litter decomposed faster or slower in stands of the same species (home field advantage; [23,24]), and monoculture litter decomposition rates were altered when the same samples were decomposed in plots that contained a mixture of stand tree species (e.g. [25]). Finally, some studies in herbaceous systems reported accelerated decomposition of standard test materials at higher plant species richness (e.g. [26]), while others found no effects of plant species but of functional group richness (e.g. [27]).

Topography (e.g. slope, aspect and relief) can affect duration and intensity of solar irradiation [28], with consequences for microclimate and thus decomposition (Fig 1e). Indeed, in climate zones with moisture limiting microbial activity, higher litter decomposition rates have been found on north facing slopes (northern hemisphere) and on lower or middle slope positions compared to south facing and upper slope locations [29]. Topography can further affect runoff, and thus soil moisture [30]. Such topography-mediated effects most likely are more important when vegetation is in early successional stages (e.g. after clear-cut) when plant canopies cannot moderate effects on microclimate as in older stands. In the longer term, topography and microrelief can affect decomposition by changing vegetation (e.g. [31,32]) and decomposer community composition (e.g. [33]; Fig 1f).

To date, little is known on the importance of stand species diversity on litter decomposition rates in forests, especially in the subtropics which are characterized by high biodiversity, warm, moist summers and cold, dry winters. Here, we present a study of decomposition rates of standard litter material of three species (*Osmanthus fragrans*, *Schima superba* and *Pinus massoniana*) contrasting in litter properties in a large-scale tree biodiversity experiment in the subtropical climate zone of East China. The study encompassed 134 plots with a planted tree species richness ranging from 0 to 24 tree species. These plots were spread over two 26 hectare sites characterized by large topographic heterogeneity. Our aims were i) to test for effects of stand species diversity (composition and richness) on litter decomposition rates, ii) to separate this effects from influences of topography conditions, and iii) to identify to which degree diversity and topographic effects are mediated by indirect microclimatic effects.

## Materials and Methods

### Study site and experimental design

**Fig 2:**
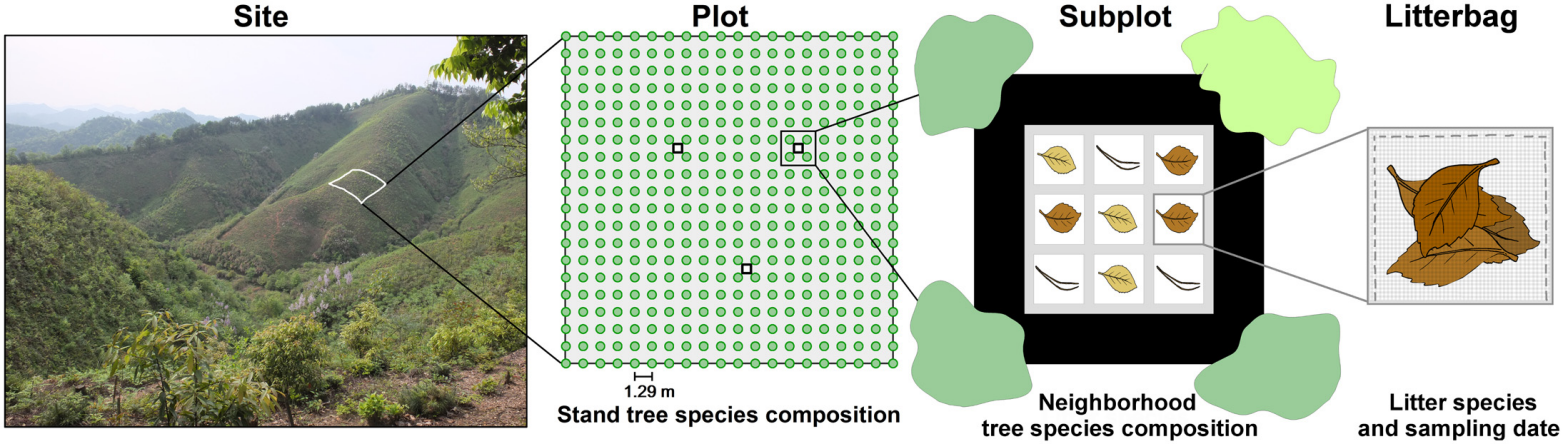
Schematic representation of the litter decomposition experiment. View on one of the BEF-China experimental sites (photograph of site A in spring 2012) and graph of one experimental plot, subplot and litterbag unit. The decomposition experiment encompassed two sites, 134 plots and three subplots per plot. Each subplot comprised three litterbags per litter species which were collected after two, six and eleven months.

The present study was conducted from Nov. 2011 to Oct. 2012 in Xingangshan, Jiangxi province, South-East China and was part of a large forest biodiversity experiment (“BEF-China”; [34,35]) established in 2009–2010 at two experimental sites (named “A” and “B”, each approximately 26 ha; see plot contour maps in [35] and Fig 2). In total, 566 plots with 400 tree individuals each arranged on a 20 × 20 planting grid (1.29 m spacing between each tree, i.e. resulting in a plot size of approx. 26 by 26 m) were established (Fig 2). For our decomposition experiment we used a subset of 134 plots, for which the species composition was obtained by successively splitting an initial pool of 16 species into equal halves until monocultures were obtained. The source species pool for this procedure differed among sites and did not overlap. By adopting this “broken stick” design, we ensured that all tree species were equally represented at each diversity level, i.e. avoided a confounding of species identity and species richness [35]. At the same time, independence of species compositions within each diversity level was maximized (no overlap). We further included communities without tree species (zero species) and with 24 species (of which 16 species were common with the lower diversity levels). This resulted in one 0-species plot, 16 monocultures, eight 2-species mixtures, four 4-species, two 8-species, one 16- and one 24-species mixture per site. Within each site, the tree-free plots were replicated three times and each community composition two times, resulting in a total of 67 plots per site. These diversity treatments were applied randomly to plots with the constraint that plots of equal composition were at least 100 m apart. Plots were weeded twice a year (spring and summer), which included cut-back of resprouting tree stumps of the former *Cunninghamia lanceolata* plantation.

Climate in this region is subtropical monsoon with a mean annual temperature of 16.7°C and around 1800 mm of precipitation. Summers are warm and moist with mean July temperatures exceeding 34°C. In contrast, winters are cold and dry with mean temperatures in January of about 1°C. Both sites are on Gneiss parent material, i.e. siliceous substrates with Cambisols and Cambisol derivates. Our experimental plots were characterized by strong topographical heterogeneity, with elevation ranging from 105–275 m a.s.l. at site A and 105–190 m a.s.l. at site B. Slope inclination averaged 30°, with a maximum of 44° [36,37].

### Litter decomposition experiment

We established three subplots within the central 9 x 9 m of each plot, which were located at fixed positions in a regular triangle with side lengths of at least 8 m (Fig 2). We placed a total of nine polyester bags (15 x 15 cm, 1 x 1.5 mm mesh size) filled with 5 g equivalent dry weight of monoculture leaf litter in each subplot (Fig 2). Three bags each contained litter from *Schima superba* Gardner & Champ. (collected in a secondary forest near the experimental sites), *Pinus massoniana* Lamb. or *Osmanthus fragrans* (Thun.) Lour. (both collected in a monoculture plantation near the experimental sites). The leaf litter had been dried at 60°C to constant weight prior to filling into bags. Overall, this design resulted in 134 plots with 64 distinct stand species compositions, 402 subplots, and 3618 litter bags (1206 per litter species).

The subplots were protected from weed ingrowth by pinning a 25 cm wide stripe of water-permeable plastic fabric around the central patch where the litterbags were placed. After two, six and eleven months, one litter bag per species and subplot was collected. This timespan was chosen because decomposition rates can best be quantified when a substantial fraction (but not all) of the litter is decomposed [21,38]. Litter was cleaned, dried at 60°C for 48 h and weighed.

No specific permissions were required for our experiment. The field study did not involve endangered or protected species.

### Environmental conditions

Topographic conditions at the plot level (mean plot aspect, inclination and solar radiation) were derived from a digital elevation model [36,37]. At the subplot level, we measured slope-aspect (separated in a north-south and east-west gradient) and microrelief curvature (ground curvature underneath the litterbags; linear scale ranging from 1 [strongly concave] to 5 [strongly convex]). Air temperature and humidity were measured every 30 minutes (HOBO Pro v2 data loggers, Onset Computer Corporation, Bourne, USA) in one subplot per tree species composition. In addition, soil moisture at 5 cm depth was measured manually once a month (ThetaProbe ML2x FD-probe, Delta-T, Cambridge, UK). The species identity and mean height of trees directly surrounding the subplots were recorded.

### Data analysis

For each subplot and species, first-order litter decomposition rate constants (k, unit: yr^-1^) were calculated by fitting X_t_ = X_0_ e^−kt^ to the data, where *X*_0_ and *X*_t_ are the litter mass at time = 0 and t, respectively. Each k-value was based on litter dry mass of at least three of the four sampling dates. Model fits were obtained on untransformed data using the ‘nls’ function of R 3.0 (R Core Team 2013).

We used analysis of variance based on linear mixed effect models to test for effects of planted tree species richness, litter species, and topography and microclimate on decomposition rate constants (ASReml 3; VSN International Ltd). The models contained the random effects planted tree species composition (the replication level for species diversity), plot (the replication level for plot topographic and microclimate data), subplot tree species composition (the replication level for the subplot species diversity), subplot (the replication level for subplot topographic and environmental data), and the interactions of litter species with composition and plot (the replication levels for the interactions of litter species with diversity and the plot-level variables; S1 Dataset). Residual distribution was inspected graphically and extreme values identified based on a residual larger than 3.5 median absolute deviations removed from the analysis (although these generally did not influence our results).

The “diversity” part of the models contained the fixed effects (in this order) ground cover (zero-diversity vs. planted plots), species richness (log-linear contrast), and a contrast indicating the presence of *Rhus chinensis*, *Cyclobalanopsis glauca* and *Castanopsis eyrei* in the plot. These species were found to explain significant amounts of systematic variation among plot species compositions. We further included litter species and its interactions with planted tree species richness in the model, i.e. tested whether the different litter types showed different effects of plot-level tree species richness. The “diversity” part at the subplot level contained the factors tree species richness (number of tree species present) of the 0–4 surrounding tree species of each subplot, the mean height of these trees and their respective species composition. When tree stumps of the former planted *Cunninghamia lanceolata* plantation were close to the subplot position and resprouting, then this species was counted to the subplot diversity of woody plants (species richness and composition).

The “topography” part of the models included, slope-aspect (decomposed into a north-south and an east-west component), altitude, plus an estimate of potential solar radiation under clear sky. To account for spatial gradients across sites that are unrelated to topography, we further added position within site (northing and easting). At the subplot level, the “topography” model contained terms for subplot slope gradient (along the two cardinal directions) and microrelief curvature of the subplot containing the respective set of litterbags.

The “microclimate” part included air temperature and humidity plus soil moisture. Temperature data were split into mean, plus daily and annual amplitudes.

We fitted two main groups of models: A “diversity-topography” model contained the “diversity” and “topographic” submodels. A “diversity-topography-microclimate” model further included the “microclimate” submodel; microclimate data was only available for half of the plots, and only for one of the three subplots, so that these analyses were only run using a subset of the total data. For both models, the percentage of variance explained by all terms was determined by fitting an equivalent linear model using classical least-squares procedures. Significances generally did not differ substantially from models fitted by REML. In the resulting fit, the percentage of total sum of squares explained by a specific term can be interpreted as amount of variance explained. The order of “diversity”, “topographic”, and, if present, also “microclimate”, was changed to all possible combinations to evaluate the extent to which microclimate is driven by the “diversity” and “topogroaphy” (see examples in [39]). Doing so allowed exploring the non-orthogonality of the different submodels. If, for example, microclimate was largely driven by topography, a large shared fraction of sum of squares would be found for the “topography” and “microclimate” submodels.

## Results

### Litter species and plot tree diversity effects

Decomposition rates were highly species-specific (*P* < 0.001). Highest decomposition rates were found for *O*. *fragrans* leaf litter with a mean k of 0.56 ± 0.08 yr^-1^ (corresponding to a mean weight loss after 11 months of 40%) followed by *S*. *superba* with 0.42 ± 0.09 yr^-1^ (32% mass loss) and *P*. *massoniana* with 0.28 ± 0.09 yr^-1^ (23% mass loss).

**Fig 3:**
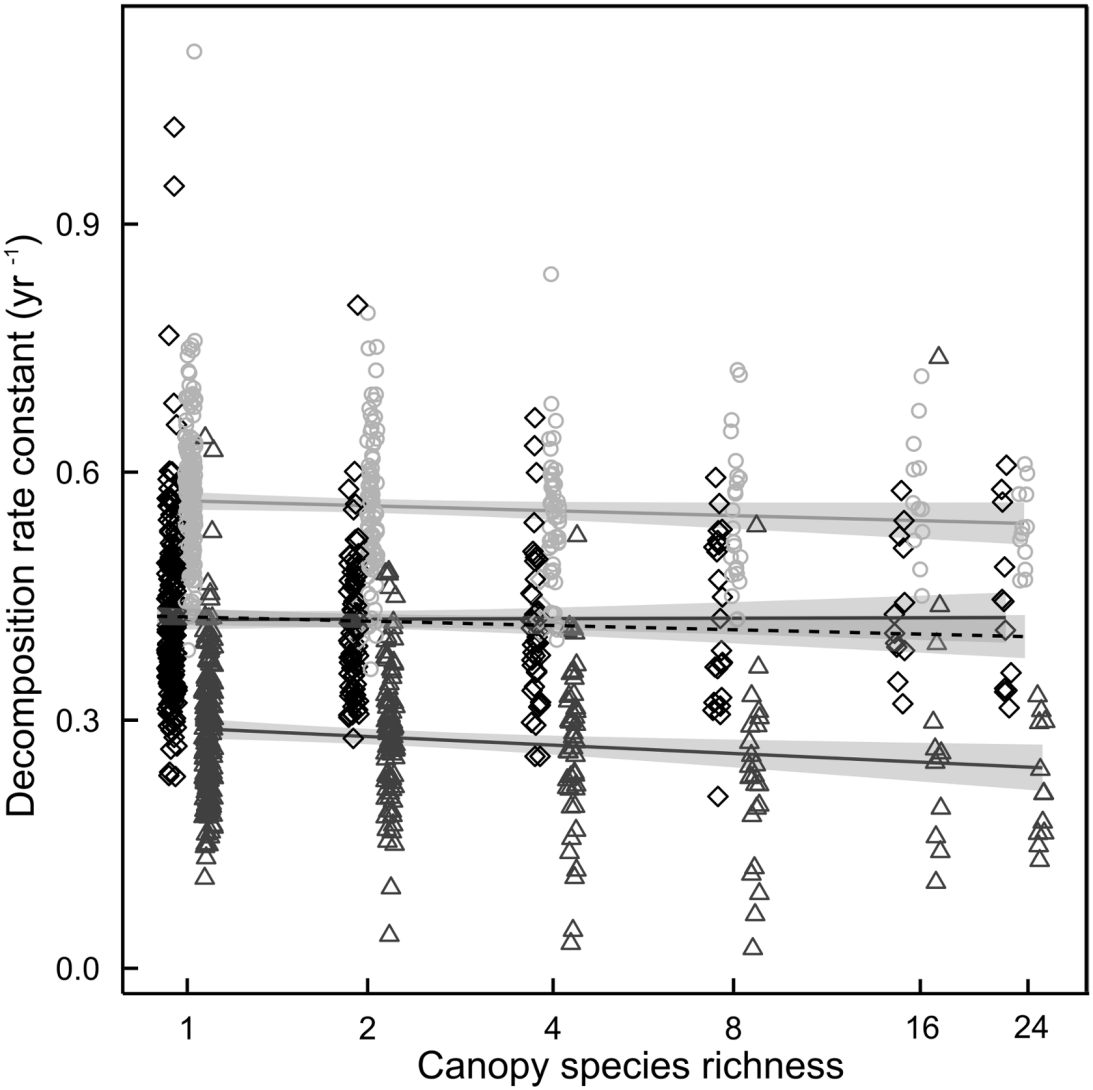
Leaf litter decomposition rate constants of *Osmanthus fragrans* (circles), *Schima superba* (squares) and *Pinus massoniana* (triangles) in dependence of stand tree species diversity. The predicted means and confidence intervals for the respective tree species are represented by the continuous lines while the mean for all three species is represented by the dashed line.

The results of our “diversity-topography” model (Tables 1–3) revealed negative effects of planted plot tree species richness on litter decomposition rate constants (*P* < 0.05; Fig 3) which however vanished in the “diversity-topography-microclimate” model, and also when diversity was tested after the presence of the species *Rhus chinensis*, *Castanopsis eyrie* and *Cyclobalanopsis glauca* within the plot level (Table 1). Diversity effects did not depend on litter species (litter species x log(richness) n.s.). However, when litter species were tested individually, decomposition rates decreased significantly with tree species richness for *Pinus massoniana* (*P <* 0.05) while *Osmanthus fragrans* showed non-significant negative and *Schima superba* positive trends. Interestingly, zero-diversity plots and plots planted with trees were statistically indistinguishable with respect to decomposition rate constants. The presence of the species *Rhus chinensis* and *Castanopsis eyrie* decreased and the presence of *Cyclobalanopsis glauca* increased litter decomposition rates (*P* < 0.05). These effects vanished in the “diversity-topography-microclimate” model with *R*. *chinensis* still showing a tendency to negative effects (*P* < 0.1). Species richness, mean height and species composition of the trees present around the subplots did not affect litter decomposition rates.

**Table 1:** Amount of variance in first-order decomposition rate constant explained by individual terms of the sequential linear “diversity-topographic model” (part 1: site and plot level).

Hierarchical level	Nominator		Denominator		Significance	Percent SS	
	Term	df	Term	ddf		P level	total
site	A vs B	1					0.0041
plot	zero-div. vs. planted pl.	1	species composition	59	n.s.	0.0	
	log(tree species richness)	1	species composition	59	*	3.4	
	presence of *Rhus chinensis*	1	species composition	59	*	4.1	
	presence of *Cyclobalanopsis glauca*	1	species composition	59	*	3.5	
	presence of *Castanopsis eyrei*	1	species composition	59	*	3.4	
	**residual species composition**	59	plot	58	n.s.	39.9	
	(sum of plot diversity part)	(64)				(54.3)	
	position within site: easting	1	plot	58	n.s.	0.4	
	position within site: northing	1	plot	58	n.s.	0.0	
	slope gradient: north-south	1	plot	58	n.s.	0.2	
	slope gradient: east-west	1	plot	58	n.s.	0.5	
	solar radiation	1	plot	58	**	5.6	
	altitude	1	plot	58	n.s.	1.0	
	(total plot topographic part)	(4)				(7.7)	
	**residual (plot)**	58				38.0	
	total	128				100.0	6.8

Topographic factors were fitted after diversity terms at the plot level. Terms are grouped by error strata (indicated in bold) and tested against the respective residual. df and ddf indicate nominator and denominator degrees of freedom of the respective F ratio. % SS indicates the percentage of sum of squares explained by each term.

*** *P* < 0.001

** *P* < 0.01

* *P* < 0.05

(.) *P* < 0.1

**Table 2:** Amount of variance in first-order decomposition rate constant explained by individual terms of the sequential linear “diversity-topographic model” (part 2: plot x litter species level).

Hierarchical level	Nominator		Denominator		Significancence	Percent SS	
	Term	df	Term	ddf		P level	total
plot x litter species	litter species	2	litter species x plot	116	***	84.8	
	litter species x (zero-div. vs. planted pl.)	2	litter species x species composition	120	n.s.	0.0	
	litter species x log(tree species richness)	2	litter species x species composition	120	n.s.	0.2	
	litter species x presence of *R*. *chinensis*	2	litter species x species composition	120	n.s.	0.1	
	litter species x presence of *C*. *glauca*	2	litter species x species composition	120	n.s.	0.1	
	litter species x presence of *C*. *eyrei*	2	litter species x species composition	120	.	0.2	
	**litter species x species composition**	120	litter species x plot	116	n.s.	6.3	
	litter species x position: easting	2	litter species x plot	116	*	0.4	
	litter species x position: northing	2	litter species x plot	116	n.s.	0.1	
	litter species x gradient: north-south	2	litter species x plot	116	n.s.	0.1	
	litter species x gradient: east-west	2	litter species x plot	116	n.s.	0.2	
	litter species x solar radiation	2	litter species x plot	116	n.s.	0.0	
	litter species x altitude	2	litter species x plot	116	n.s.	0.1	
	**residual (litter species x plot)**	116				7.5	
	total	260				100.0	72.3

Topographic factors were fitted after diversity terms at the plot level. Terms are grouped by error strata (indicated in bold) and tested against the respective residual. df and ddf indicate nominator and denominator degrees of freedom of the respective F ratio. % SS indicates the percentage of sum of squares explained by each term.

*** *P* < 0.001

** *P* < 0.01

* *P* < 0.05

(.) *P* < 0.1

**Table 3:** Amount of variance in first-order decomposition rate constant explained by individual terms of the sequential linear “diversity-topographic model” (part 3: subplot level).

Hierarchical level	Nominator		Denominator		Significance	Percent SS	
	Term	df	Term	ddf		P level	total
subplot	tree species richness	1	subplot tree composition	77	n.s.	0.0	
	mean tree height	1	subplot tree composition	77	n.s.	0.2	
	**subplot tree composition**	77	subplot	154	n.s.	29.3	
	(total subplot diversity part)	(79)				(29.5)	
	slope gradient: north-south	1	subplot	154	n.s.	0.6	
	slope gradient: east-west	1	subplot	154	n.s.	0.0	
	microrelief curvature	1	subplot	154	n.s.	0.0	
	(total subplot topographic part)	(3)				(0.6)	
	**residual (subplot)**	236				69.9	
	total	319				100.0	10.5
subplot x litter species	**residual (subplot x litter species)**	462					10.4
total		1170					100.0

Topographic factors were fitted after diversity terms at the plot level. Terms are grouped by error strata (indicated in bold) and tested against the respective residual. df and ddf indicate nominator and denominator degrees of freedom of the respective F ratio. % SS indicates the percentage of sum of squares explained by each term.

*** *P* < 0.001

** *P* < 0.01

* *P* < 0.05

(.) *P* < 0.1

### Topography

When analysing the full data set, plot-level topographic effects increased with potential solar radiation (*P* < 0.01 for radiation; Table 1). Analysis by litter species showed positive responses of *O*. *fragrans* and *P*. *massoniana* to increasing solar radiation (P < 0.05) while no effects were found for *S*. *superba*.

When analysing only the plots with microclimate data loggers, south and west facing slopes and potential solar radiation increased litter decomposition. These effects were significant at *P* < 0.01, *P* < 0.05 and *P* < 0.05 when fitted before microclimate and at *P* < 0.05, *P* < 0.1 and *P* < 0.05 when fitted after microclimate, respectively.

### Microclimate

Decomposition rates increased with mean annual air temperature amplitudes (*P* < 0.05) and annual soil moisture means (*P* < 0.01) affected decomposition rate constants positively while mean daily temperature amplitudes (*P* < 0.01) had a negative effect when fitted before diversity and topography in the model. When fitted after diversity, the significance of mean annual temperature amplitudes and soil moisture decreased to P < 0.1 and P < 0.01, respectively. If fitted after diversity and topography, only soil moisture remained significant (P < 0.001). Mean annual soil moisture and daily temperature amplitudes were strongly affected by topographical factors (aspect gradients and solar radiation *P* < 0.01, altitude *P* < 0.1).

### Interdependence of diversity, topography and microclimate models

**Fig 4:**
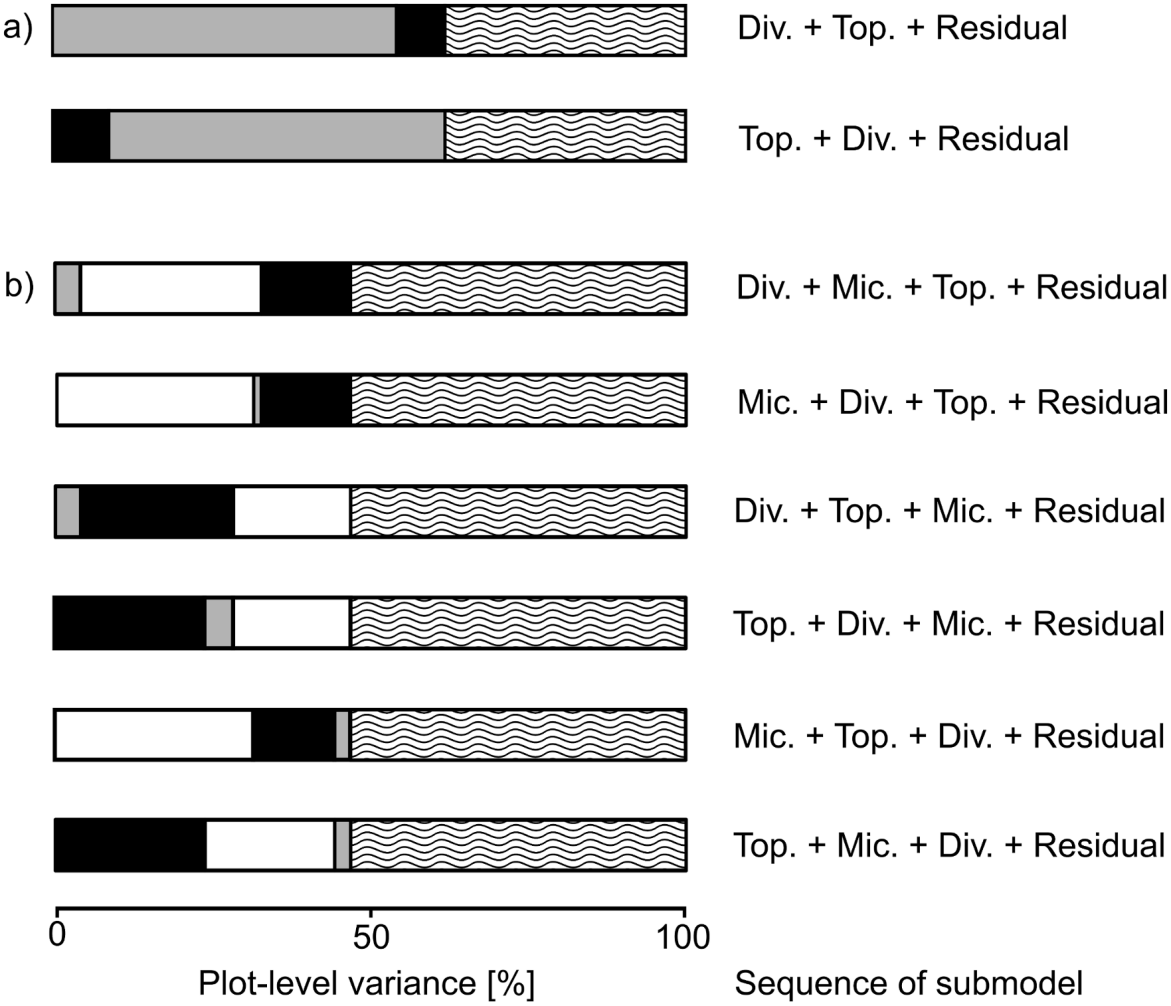
Fraction of plot-level variance explained by diversity (Div.), topography (Top.), and microclimate (Mic.). The explained variance by the submodels is shown for all possible orders in the sequentially fitted (a) “topography”-model (species composition is included in Div.) and the (b) “microclimate”- model (species composition cannot be separated from the plot residual). While diversity and topography submodels are largely independent, reduced amounts of explained variance when microclimate was fitted first suggests that their effects were mediated by microclimate.

In accordance with the large differences between litter species, the largest amount of variation occurred at the litter species × plot level (Table 2). At the plot level (Table 1), the diversity model explained much more variation than topography (54.3% vs. 7.7%)–this remained true even when plot species composition, which contained a large component of the random between-plot variation, was not considered part of the diversity model (14.4% vs. 7.7%). Tree species richness explained about 3.4% of the plot-level variation, and the presence of the three tree species *R*. *chinensis*, *C*. *glauca* and *C*. *eyrie* each explained an additional 3–4%. Tree species composition explained the largest component at the plot level with 39.9%. Computed potential solar radiation was the dominant topographic determinant of decomposition rate constants (5.6% of plot-level variation). Plot-level diversity and topography effects were relatively independent, i.e. results did not depend on the order in which the submodels were fitted (Fig 4). At the plot × litter species level (Table 2), only litter species (85%) and the interaction of litter species with tree species composition (6.4%) explained more than 1% of the variance. At the subplot level (Table 3), the size and diversity of the trees surrounding the subplot did not explain any relevant amount of variation; in contrast, a large component of the random between-subplot variation was explained by the specific composition of the set of trees around the subplot.

Diversity, topography, and microclimate submodels explained varying fractions of total variation, depending on their order. In particular, microclimate, when fitted first, reduced the amount of variation that was explained by the diversity or topography submodel, respectively; conversely, diversity and topography explained 8.9 and 34.4% of the variance explained by microclimate (31.4%; Fig 4). The tree species *R*. *chinensis* explained the biggest fraction of variance in the diversity submodel (3.3% of the plot level variation), when fitted before microclimate. The explained variance of this species decreased to 1% when the diversity submodel was fitted after microclimate. Soil moisture as the main microclimatic explanatory factor for litter decomposition rates was mediated by species diversity by around 11%.

## Discussion

Our analysis demonstrates that stand tree species diversity, site topography, and microclimate explain important amounts of variation in litter decomposition rate constants of the investigated litter species (Fig 1c–1f and 1i). Comparison of models differing in the order of the fitted factors suggested that stand species diversity and topographic effects were at least in part mediated by microclimatic changes (Fig 1d and 1e). Tree species composition explained the largest amount of variance at the plot level. Differences in species properties such as tree height or the degree of canopy closure may have affected microclimate but also detritivore abundances and their activity. Although the effect size was small, stand tree species richness reduced decomposition rates, which was connected to slightly lower soil moisture in species rich plots and the presence of certain tree species which may either indicate species complementarity or species sampling effects [40].

Our findings indicate that stand tree species richness can reduce leaf litter decomposition rates already in early stages of stand development although effects sizes were relatively small (Fig 1c and 1d). So far, no effects of stand species richness on decomposition rates of standard litter material were found in forests (e.g. [19–21]). Other authors report positive effects of tree stands with 2-species mixtures on monoculture decomposition rates of spruce litter [25] and positive effects of plot-specific litter mixtures compared with monoculture decomposition rates [21]. Given that diversity effects (e.g. resource capture, biomass production) usually increase with time after establishment of new experimental communities [41], especially for soil mediated processes, the initial effects we observed may well grow over the next years. To our knowledge, it has yet not been analysed whether diversity effects on decomposition actually change with stand development. However, litter decomposition rates indeed change with the successional stage of forests in tropical dry [42] and in subtropical forests [21], suggesting that decomposition processes are highly dynamic in time due to changes of the biotic and abiotic environment.

It is important to separate effects of stand species richness (Fig 1c and 1d) from effects of litter species richness (Fig 1a). The first has been investigated quantifying decay rates of standard materials in forest stands differing in tree species richness. The second has been tested by decomposing different litter mixtures in homogeneous environments. When both stand and litter diversity change concomitantly (e.g. [2,21]), effects of these two factors are hard to disentangle.

The three litter species we investigated differed in decomposition rates. Litter chemistry often determines decomposition rates, with slower decomposition at lower nitrogen concentrations. In our study, C:N ratios indeed explain the observed ranks in decomposition rates (*Osmanthus fragrans*: C/N = 46.9; [own data] > *Schima superba*: C/N = 59.6; [21] > *Pinus massoniana*: C/N = 74.3; [21]). However, other, possibly correlated, characteristics such as lignin [43], total phenolic or tannin content [44,45], and non-aromatic labile carbon compounds such as non-structural carbohydrates [46] may also have been relevant. Leaf structural difference may also have affected the litter’s water holding capacity, which in turn might have altered microbial activities and decomposition [47]. In our study, *S*. *superba* leaves were comparatively flat and stuck together in layers, which might trap moisture for prolonged periods. *O*. *fragrans* leaves were stiff and corrugated, which might have led to faster desiccation due to the large inter-leaf spaces, and the same might have happened with the needle structure of *P*. *massoniana* leaves.

In our experimental design, tree species composition was replicated only in two separate plots. Naturally, the variance explained by the composition term contained a substantial fraction of the random between-plot variation; indeed, the variance component for mixture estimated by the REML fit was close to zero, indicating that mixture effects were small after accounting for the specific effects of the presence of a few important species. Tree species differed massively in growth rates, at least in the initial stages of stand development during which we conducted our study. After two and three years, *Choerospondias axillaris* and *Nyssa sinensis* monoculture stands already started to close their canopy, whereas slow-growing species such as *Castanopsis eyrei* or *Cyclobalanopsis glauca* (cf. [44,48]) barely shaded our litter bags. Our analysis indicated significant effects of the presence of particular species; however, the identified effects of the species are difficult to interpret because particular species co-occur in many plots as consequence of the “broken stick” design; their effects thus cannot fully be separated. Nevertheless, the most important single species we found were of low productivity (cf. [48]) and had below-average decomposition, i.e. they seemed to be exceptions from the overall trend of decreased decomposition at high diversity (i.e. at higher leaf area index; cf. [49]). One reason might be that the cover in these plots was so low that high temperatures or desiccation slowed decomposition. The fact that the variance explained by species diversity dropped when fitted after the microclimatic covariates suggests that diversity effects were at least partly mediated by climate.

The tendency of lower soil moisture in more diverse mixtures may have resulted from increased water use due to higher biomass and larger transpiration [50], or due to higher interception in high diverse mixtures. Data from Peng et al. [49] indeed show positive diversity effects on canopy leaf area in the same plots two (site A) and three (site B) years after our study. We do not have data from previous years, but this trend could already have been apparent at the time of our experiment. However, we can only speculate about the underlying effects since especially site topography can strongly affect soil water regimes [51]. Other authors found either no effects (e.g. [52]) or increases of soil moisture contents along diversity gradients (e.g. [53,54]). However, these studies were conducted in grassland on flat areas and in different climate zones, which are difficult to compare to the present study. Further, diversity effects on litter decomposition may also be mediated by shifts in decomposer community composition, which however, were not captured by our experiment.

Although controlling litter decomposition rates, neither soil moisture nor temperature amplitudes were significantly affected by canopy species richness. Especially topography (which cannot be separated from the plot residual in our experimental design) but also tree species composition appear to be the main drivers of microclimatic effects at this early stage of the experiment. Topographic effects on litter decomposition rates were small compared to the larger effects of tree species richness. However, topography strongly affected mean daily and annual temperature amplitudes. The main underlying driver was slope orientation, but surrounding hills also shaded some plots early in the morning and in the later evening. These results underline the importance of stand tree species diversity and environmental factors for litter decomposition rates especially in young forest stands and the need to implement them in the respective statistical models. However, in our study, decomposition rates were less sensitive to temperature and rather driven by soil moisture. This may have been the case because temperatures generally were high at the field site, and temperature and moisture often are negatively correlated–a potentially accelerating effect of higher temperatures on decomposition is then masked by a decelerating effect of drying.

## Conclusions

Our study is one of the first to compare direct topographic and tree species diversity effects on litter decomposition rates from microclimate-mediated indirect effects of these terms. However, the data we present cover early stages of stand development (two to three years) and the implications for more mature stands may be different. Above all, we propose that the importance of topographic factors will decrease over time while tree species diversity effects become more important as canopy development progresses. Our data suggest that, among the microclimatic factors investigated, soil moisture is particularly important. The available literature indicates that diversity effects on soil moisture can be positive or negative, depending on the hydrological processes dominating, and the effects we found may therefore well change in the future. In the long term, tree species diversity may also cause changes in soil physico-chemical properties, incl. acidity, bulk density, nutrient availability, and presence of secondary compounds, which might lead to additional effects that are mediated by changes in decomposer community composition and activity [21,42,55].

## Supporting Information

S1 Dataset Litter decomposition data presented in this article. (ZIP)
